# A qualitative exploration of how nurses experience autonomy in professional roles

**DOI:** 10.1186/s12912-025-04217-z

**Published:** 2025-12-11

**Authors:** Gizem Açıkgöz, Ülkü Baykal

**Affiliations:** 1 Nursing Department, Faculty of Health Sciences, İstanbul Kent University, Cendere Street No:24, Kâğıthane/İstanbul, 34406 Türkiye; 2 Nursing Department, Faculty of Health Sciences, Altınbaş University, Mahmutbey, Dilmenler Street No:26, Bağcılar/İstanbul, 34217 Türkiye

**Keywords:** Autonomy in nursing, Professional autonomy, Nursing roles, Qualitative study, Nursing practice, Phenomenology

## Abstract

**Background:**

Autonomy in nursing refers to the ability to make independent decisions in professional practice, in accordance with fundamental principles, practice standards, and legal regulations. Although autonomy has been widely studied for many years, little is known about how it is experienced within the framework of specific professional roles. Focusing on roles provides a more concrete reflection of clinical practice and offers unique insights that extend beyond general definitions of autonomy.

**Methods:**

This qualitative phenomenological study was conducted with 17 nurses working in different hospital settings. Participants were selected using purposive and snowball sampling to ensure diversity. Data were collected through online semi-structured interviews. Analysis followed Colaizzi’s seven-stage content analysis method, supported by MAXQDA software, and was guided by the COREQ checklist to enhance rigor.

**Results:**

The analysis revealed four themes: professional autonomy, professional autonomy in nursing, individual professional autonomy, and nursing role autonomy. Professional autonomy was found to be closely linked to professional regulations, professionalism, independence, and responsibility. While both positive and negative factors influenced their autonomy, many aspects were identified as areas with potential for improvement. Nurses demonstrated varying levels of autonomous behavior across different professional roles.

**Conclusion:**

Nurses show considerable awareness of professional autonomy, yet their practice is shaped by both internal and external factors. They sustain and strengthen their autonomy through professional collaboration, reflective practice, and continuous learning, while systemic support from health managers and policymakers is also required to establish enabling structures. These findings highlight role-based autonomy as a critical dimension of nursing practice, with implications for professional development, organizational management, and health policy.”

**Trial registration:**

Not applicable.

## Backround

Autonomy refers to individuals’ ability to make decisions about themselves based on their values. Professionally, it refers to the independence of a profession from other professions, both conceptually and practically. It is also defined as the control of professional decisions by members of the profession [1, 2]. Professional autonomy in nursing involves independent decision-making in line with basic principles, practice standards, and legal regulations related to the profession [3–5]. One of the most basic requirements of professional development is nurses’ ability to make autonomous decisions within their professional authority, monitor the results of their decisions, and take responsibility for said results [6–8].

The development of professional expertise in nursing, which is defined as the integration of theoretical knowledge gained through professional education, clinical experience, and professional judgment, has increased the authority and responsibilities of the nursing profession, expanding its independent roles and functions. In addition to the foundational roles of care, education, research, management, decision-making, and patient advocacy, contemporary nursing roles also include communication and coordination, rehabilitative and therapeutic roles, career development, autonomy and responsibility, and advisory roles [7, 9–12]. These roles contribute to the development and professionalization of nursing, requiring nurses to make autonomous decisions related to their professional roles. In addition to influencing the nursing profession itself, nurses’ autonomy plays an important role within the health system, significantly impacting patient care, organizational effectiveness, and overall healthcare outcomes through improved decision-making and continuity of care [10, 13].

The effects of nurses’ autonomy in the health system can be addressed in different fields. Professional autonomy in nursing enhances clinical outcomes related to the care of individuals, ensures the continuity and safety of nursing care, and improves the quality of nursing care [2, 4, 6, 8]. Moreover, it contributes to the prevention of complications, shorter hospital stays, and positive outcomes for patients, enhancing cost-effectiveness for hospital organizations. At the same time, it increases nurses’ control over healthcare practices such as care planning, patient monitoring, clinical decision-making, and participation in organizational and policy-related processes, thereby developing confidence and professional satisfaction [8, 14]. Nursing autonomy has been extensively studied in Türkiye and internationally, demonstrating its close relationship with professional regulations, standards of practice, and legal frameworks, as well as its influence on clinical outcomes, quality of care, job satisfaction, and professional development [1, 2, 4, 5, 8, 14, 15]. However, despite this extensive body of research, little attention has been paid to how autonomy is experienced across specific professional roles in nursing, which provides a more concrete reflection of clinical practice and carries important implications for health systems and nursing practice worldwide [10, 16, 17].

This study offers novel insights by addressing autonomy through the lens of professional roles, highlighting dimensions that are often overlooked in general discussions of autonomy, with implications not only for nursing practice but also for health managers, policymakers, and nurses themselves in strengthening the organization of health systems.

### Research questions

1. How do nurses conceptualize and experience autonomy within their professional roles?
2. Which internal and external factors shape nurses’ professional autonomy?
3. In what ways do nurses sustain or strengthen their autonomy in the face of professional challenges?

## Methods

### Study design

This study employed an interpretive phenomenological qualitative design, grounded in Heidegger’s hermeneutic philosophy, to explore how nurses construct and interpret the meaning of autonomy within their professional roles. The aim was not only to describe the lived experience but also to understand the contextual and structural factors shaping that experience. Although Colaizzi’s seven-step method provided the analytic framework, interpretive attention was given to how participants constructed and understood autonomy within their professional and institutional contexts. Throughout the research process, the authors maintained reflexive awareness of their assumptions to ensure coherence between philosophical orientation and analysis. The study followed the Consolidated Criteria for Reporting Qualitative Research (COREQ) guidelines to ensure rigor and transparency in reporting [18–21].

### Participants and settings

The study was conducted with 17 nurses employed in university, public, and private hospitals in Istanbul, Türkiye, between September 2021 and January 2022. Participants were recruited through maximum variation purposive sampling combined with snowball sampling to ensure diversity in age, education level, professional experience, clinical unit, and institutional setting. Initially, participants were purposefully selected to represent different hospital types and clinical settings. Nurses with less than two years of professional experience were excluded, considering Benner’s (1984) “From Novice to Expert” theory, which suggests that nurses typically develop professional competence and autonomous practice after 18–24 months of clinical experience. Subsequently, additional participants were identified through snowball referrals; however, only those recommendations that contributed to sample diversity were included. Although gender diversity could not be fully achieved, variation across other characteristics was ensured [22].

### Inclusion criteria

- Actively employed in a university, public, or private hospital in Istanbul.
- Member of the Turkish Nurses Society (TNS).
- Holding at least a bachelor’s degree in nursing.
- Having a minimum of two years of professional experience.

### Exclusion criteria

- Nurses without a bachelor’s degree or with less than two years of professional experience.
- Individuals not meeting the TNS membership criterion.
- Those unwilling to participate or unable to complete the interview process.

### Data collection

The semi-structured interview guide consisted of eleven open-ended questions designed to explore nurses’ perceptions and experiences related to professional autonomy. The questions were arranged sequentially to move from general understandings of professional autonomy to autonomy in nursing practice, and finally to autonomy within specific professional roles, allowing participants to articulate both conceptual and experiential dimensions of autonomy (Table 1). Each interview lasted 25–41 min, with an average duration of 31 min. All interviews were conducted via an online meeting program and audio-recorded with participants’ permission. Field notes were taken to capture contextual observations and nonverbal cues throughout the sessions.

**Table 1:** Semi-structured interview questions

Informed Consent	Dear Participant, This study aims to explore the scope of nurses’ autonomous behaviours related to their professional roles, the factors influencing these behaviours, and suggestions for strengthening professional autonomy. Data will be collected through in-depth individual interviews, a qualitative research method. Ethical approval and institutional permission have been obtained. Participation in the study is entirely voluntary, and each interview will take approximately 30 to 60 min. With your permission, the interview will be recorded; If you do not consent to recording, the researcher will take brief notes during the interview. All information obtained will be used solely for research purposes, and your personal and institutional data will remain confidential. You may choose not to participate or withdraw from the study at any time without any consequences. If you wish to receive the study results after completion, please inform the researcher at the end of the interview. Based on the information provided above, do you agree to participate in this research?
Interview Questions	1. Could you briefly introduce yourself? (Education, work experience, position, institution.) 2. How do you define professional autonomy, and what does it mean to you? 3. What behaviors do you think fall within the scope of professional autonomy in nursing? 4. Which concepts do you consider related to professional autonomy? (e.g., professionalism, norms, job descriptions) 5. How would you describe nurses’ current level of autonomy in Türkiye? 6. What main challenges do nurses face in demonstrating professional autonomy? 7. What factors influence nurses’ ability to act autonomously? 8. How should autonomy be demonstrated within the following nursing roles? o Caregiver role o Training role o Research role o Administrative role o Decision-making role o Patient advocacy role o Communication and coordination role o Rehabilitation role o Comforting role o Therapeutic role o Career-enhancing role o Autonomy and responsibility role o Consultancy role 9. What key elements should be considered when demonstrating autonomy in nursing roles? 10. How do you evaluate your own professional autonomy, and what factors influence it? 11. Is there anything you would like to add that was not covered in the interview?

**Table 2:** Data analysis process using the Colaizzi seven-step method

Step No	Step	Implementation
1	Familiarization	The data was transcribed after the interviews. To become familiar with the data, the recordings were reviewed twice, and the texts were read thrice.
2	Identifying significant statements	Data were examined using the MAXQDA 20 program, words and word groups were identified in accordance with the research topic and purpose, and relevant quotations were selected.
3	Formulating meanings	The selected quotations were converted into codes appropriate to the research phenomenon, and a code list consisting of 103 codes was recorded.
4	Clustering themes	The codes obtained were combined into sub-themes and then into main themes according to their similarities and relationships. Four main themes and 22 sub-themes were identified.
5	Developing an exhaustive description	A complete and comprehensive description of the phenomenon was written with the selected codes and themes.
6	Producing the fundamental structure	Explanations were condensed into a short and compact statement covering the aspects considered essential to the phenomenon’s structure.
7	Seeking verification of the fundamental structure	The structure created was conveyed to five participants to be evaluated in terms of accuracy of the data and compatibility of meaning with the code, and feedback was received.

**Table 3:** Characteristics of the participants

Participant	Age	Gender	Education	Hospital	Department	Professional Experience
Nurse 1	32	Female	Master	Public	Neonatal ICU	10
Nurse 2	35	Female	PhD	Private	Nursing Administration	14
Nurse 3	32	Female	Master	University	Educational Nurse	9
Nurse 4	38	Female	Master	Private	Supervisor	16
Nurse 5	32	Female	Master	Private	General ICU	10
Nurse 6	26	Female	Bachelor	Public	General ICU	5
Nurse 7	25	Female	Bachelor	Public	CVS ICU	4
Nurse 8	27	Female	Master	Public	Women’s Health Service	5
Nurse 9	31	Female	Master	Private	Oncology Service	9
Nurse 10	27	Female	Bachelor	University	Reanimation ICU	5
Nurse 11	36	Female	Bachelor	Public	Emergency Service	14
Nurse 12	36	Female	Master	Public	Adult ICU	15
Nurse 13	32	Female	Master	Public	Chest Disease Service	8
Nurse 14	34	Female	Bachelor	Public	General Surgery Service	12
Nurse 15	25	Female	Bachelor	University	Infection Disease Service	2
Nurse 16	37	Female	PhD	Public	Cardiology ICU	15
Nurse 17	27	Female	Master	Public	Outpatient Service	5

**Table 4:** Themes and sub-themes of the research

Theme	Descriptions	Sub-Theme	Brief Description	Illustrative Quote
Professional Autonomy	Reflects nurses’ general understanding of autonomy as independence, responsibility, and professional judgment.	Concept of professional autonomy	Nurses framed professional autonomy as acting within job descriptions and professional principles, in line with practice standards and legal regulations; autonomy is realized when independent decisions are made “under these written rules.”	*“We have different areas of practice, and each has its own […] we make our own decisions under these written rules.”* Nurse 15
		Related concepts of professional autonomy	Autonomy was closely linked to professionalism, professional regulations, independence, responsibility, and continuous education/development; participants often described professionalism and autonomy as mutually reinforcing.	*“Professionalism is as important as autonomy for advancing […] they even complement each other… one cannot exist without the other.”* Nurse 6
Professional Autonomy in Nursing	Describes how autonomy is experienced specifically within the nursing profession and practice, emphasizing professional standards and legal frameworks.	Frameworks of professional autonomy in nursing	Nurses described autonomy as grounded in the nursing process, where assessing needs, planning, implementing, and evaluating care represent its basic framework.	*“Professional autonomy is implemented within the nursing process. I independently assess patient needs, plan, apply, and then evaluate the outcomes.”* Nurse 7
		Factors affecting nurses’ professional autonomy	Education, professional development, teamwork, and managerial support were seen as key factors enhancing autonomy, while workload and hierarchical culture limited it.	*“Education, professional development, teamwork, and managerial support were seen as key factors enhancing autonomy, while workload and hierarchical culture limited it.”* Nurse 9
		The main problems in nursing professional autonomy	Weaknesses of professional organizations, physician dominance, and unclear regulations were identified as major barriers to autonomy.	*“One of the biggest obstacles is that our professional organizations are weak, so our voices are not strong enough to protect our autonomy.”* Nurse 12
		Professional autonomy of nurses in Türkiye	Most nurses considered autonomy in Türkiye as moderate, often constrained by cultural and institutional hierarchies.	*“In Türkiye, our autonomy is still not at the desired level. We often wait for physician approval even in areas where we should act independently.”* Nurse 5
		Issues to be considered in the professional autonomy of nurses	Participants stressed the importance of legal clarity, stronger professional organizations, and better working conditions to support autonomy.	*“Participants stressed the importance of legal clarity, stronger professional organizations, and better working conditions to support autonomy.”* Nurse 14
Individual Professional Autonomy	Represents nurses’ self-awareness and confidence in making independent professional decisions.	Self-assessment of professional autonomy	Nurses evaluated their own autonomy as moderate, emphasizing that their ability to act independently often depends on confidence, experience, and support from colleagues.	*“I think my autonomy is moderate. I can decide on many things, but sometimes I hesitate, especially when I feel my decisions may not be accepted by others.”* Nurse 8
		Factors affecting professional autonomy	Motivation, interest in the profession, self-esteem, and continuous professional learning were identified as personal factors influencing autonomy, while institutional barriers limited its realization.	*“When I am motivated and keep learning, I feel strong enough to make my own decisions. But sometimes organizational obstacles make me step back.”* Nurse 4
Autonomy Regarding Nursing Roles	Highlights how autonomy manifests in specific nursing roles, such as care, education, management, and research, revealing contextual differences among roles.	Caregiver role	Autonomy in caregiving involved independently assessing patient needs, planning holistic care, and taking responsibility for outcomes.	*“Autonomy in caregiving involved independently assessing patient needs, planning holistic care, and taking responsibility for outcomes.”* Nurse 7
		Training role	Nurses describe autonomy in deciding the scope, method, and content of patient education.	*“Nurses described autonomy in deciding the scope, method, and content of patient education.”* Nurse 3
		Research role	Staying updated with scientific evidence and incorporating research findings were considered core to professional autonomy.	*“I follow new research and apply it in my daily practice without waiting for directives.”* Nurse 6
		Administrative role	Autonomy in administration included advocating for colleagues, organizing workflow, and making team decisions.	*“As a manager, I sometimes have to defend my team’s choices and make independent decisions for the unit.”* Nurse 14
		Decision-making role	Nurses emphasized autonomy in clinical decision-making, especially when patient safety was at stake.	*“In urgent situations, I must make decisions quickly and independently to protect the patient.”* Nurse 11
		Patient advocacy role	Nurses took autonomous action to protect patients’ rights and interests, even when this meant opposing others.	*“Sometimes I stand against doctors or relatives if it’s necessary to protect my patient’s rights.”* Nurse 5
		Communication and coordination role	Effective communication with other professionals and coordinating care were perceived as part of autonomy.	*“Ensuring coordination in the team and voicing my opinion makes me feel truly autonomous.”* Nurse 1
		Rehabilitation role	Nurses exercised autonomy in identifying patients’ rehabilitation needs and mobilizing multidisciplinary support.	*“When I see my patient needs extra support, I decide to involve physiotherapy or another branch.”* Nurse 12
		Comforting role	Autonomy included independently providing psychological and emotional support to patients.	*“Sometimes, comforting a patient is my own decision, and I see it as part of my professional independence.”* Nurse 16
		Therapeutic role	Nurses monitored treatment effects and made independent choices about next steps in care.	*“If the treatment doesn’t go as expected, I decide what supportive action should be taken.”* Nurse 9
		Career-enhancing role	Autonomy was also viewed as creating and pursuing career development opportunities.	*“I don’t wait for opportunities; I create them myself to improve my career.”* Nurse 4
		Autonomy and responsibility role	Independent decision-making was tied to the responsibility of ensuring continuity of care.	*“Autonomy is about taking responsibility so care continues without disruption.”* Nurse 17
		Consultancy role	Nurses provided independent counseling to patients and families beyond illness care.	*“I advise patients not only about their illness but also about protecting and maintaining health.”* Nurse 10

Initially, five volunteers suitable for this study’s sample criteria were contacted, interviews were conducted, and these individuals referred to 12 additional volunteers, resulting in a total of 17 participants. Potential participants were contacted via e-mail, and all interviews were conducted online due to pandemic-related restrictions. Before each interview, participants were informed about the study via e-mail and provided verbal informed consent at the beginning of the online session.

Data collection continued until data saturation was reached. Saturation was determined when no new meanings or potential codes emerged from the interviews, and previously identified categories were consistently repeated and confirmed across participants. The research team continuously reviewed transcripts during the analysis process, comparing new data with existing interpretations to ensure that no additional insights or variations in meaning were identified. This process confirmed that the sample size was sufficient to capture the depth and range of experiences relevant to the phenomenon under investigation [22].

### Data analysis

The MAXQDA 20 program was used to analyze the study’s data, using the content analysis method. This method aims to categorize the data under codes, subthemes, and themes [22]. The study adhered to Colaizzi’s seven-step method to evaluate the data, with results appearing in Table 2 [19].

### Ethical considerations

Ethical approval for the research was obtained from a public university ethics committee and institutional permission was obtained from the Turkish Nurses Association Istanbul branch. The study’s purpose and scope were explained to the participants, and participants were informed that the data obtained from the research would be kept confidential and would be used only within the scope of the research. Informed consent of participation was obtained in writing before online interviews.

### Rigor

To ensure the trustworthiness of the study, several strategies were employed in line with the criteria of credibility, dependability, confirmability, and transferability. Triangulation was achieved by including participants from different hospital settings, units, and levels of experience to capture diverse perspectives. Peer debriefing with qualitative research experts supported the interpretation process and enhanced analytic rigor. Member checking was conducted with five selected participants identified in collaboration with the Turkish Nurses Society (TNS) secretariat. They were asked to consider this criterion when suggesting colleagues for the study, and all TNS memberships were verified again in coordination with the Society to ensure participant eligibility. An audit trail and analytic memos were maintained throughout the research process to ensure transparency in methodological decisions. In addition, two researchers independently coded the data and reached consensus through discussion, which further strengthened the dependability and confirmability of the findings.

### Findings

#### Characteristics of the participants

The participants were all female nurses with an average age of approximately 31 years. More than half held a master’s degree, and their average professional experience was around nine years. Most of the nurses worked in public hospitals, while others were employed in university and private settings. The participants represented a diverse range of clinical units (Table 3).

### Interview results

Evaluation of the data collected from the online interviews resulted in four main themes (“professional autonomy,” “professional autonomy in nursing,” “individual professional autonomy,” and “nursing role autonomy”) and 22 sub-themes (Table 4).

### Professional autonomy

Nurses defined professional autonomy using two basic approaches: the scope of the concept and other related concepts.

### Concept of professional autonomy

Nurses stated that professional autonomy involved adhering to professional rules, being independent and competent, and advocating for the profession. They emphasized that decision-making based on professional rules and legal regulations forms the basis of professional autonomy.

One nurse commented: *“We have different areas of practice, and each has its own professional knowledge and professional rules according to law. We act autonomously when we make our own decisions under these written rules.” (Nurse 15).*

### Related concepts of professional autonomy

Nurses stated that they considered professionalism, professional regulations, ethical behavior, responsibility, awareness, leadership, and independence to be related to the concept of professional autonomy. They emphasized that professionalism is closely related to autonomy because it requires professional training and development.

One nurse stated that *“professionalism is as important as autonomy for advancing a profession; they even complement each other… one cannot exist without the other” (Nurse 6).*

### Professional autonomy in nursing

Nurses defined five basic components of professional autonomy in nursing: frameworks of professional autonomy in nursing, factors affecting nurses’ professional autonomy, the main problems in nurses’ professional autonomy, professional autonomy of nurses in Türkiye, and issues to be considered in nurses’ professional autonomy.

### Frameworks of professional autonomy in nursing

Nurses stated that nursing care, treatment practices, education, research, career development, counseling, and patient advocacy constitute the scope of professional autonomy in nursing, emphasizing that nurses should be able to evaluate the patient in line with the nursing process, determine patient needs, and plan/implement appropriate interventions.

*When you refer to professional autonomy*, said one nurse, *the first thing that comes to mind is nursing care behaviors. We have the right to take initiative…according to the patient’s needs (Nurse 2).*

### Factors affecting nurses’ professional autonomy

Nurses stated that the approach to the autonomy of the profession, the specificity of professional regulations and awareness of them, personal and professional development, managerial approach and policies, professional associations, role models, working conditions, and reward systems are all factors that affect professional autonomy in nursing. They stated that professional qualifications and self-improvement affect nurses’ professional autonomy.

On this subject, one nurse commented: *“Level of education and development is especially important. The more competent the person is in the field and the more competent they feel, the more confident and self-assured they will be in providing service or care. They will make better decisions and act more autonomously” (Nurse 3).*

### The main problems in nurses’ professional autonomy

Nurses stated that the main problems they experienced in demonstrating professional autonomy involved social attitudes towards the nursing profession, poor working conditions, negative attitudes of management, inadequate professional development and organization, lack of contact with academics in the field, ignorance of legal rights, and uncertainties in legal regulations. Nurses also highlighted inadequacies in the work of nursing professional organizations, with one participant making the following comment:

*I think the lack of commitment to each other as colleagues is an important problem. In terms of professional behavior, we sometimes see biases, even among people working in the same unit. The work of professional organizations is not sufficient. Of course, the lack of membership in organizations is another problem” (Nurse 13).*

### Professional autonomy of nurses in Türkiye

Nurses expressed different views on the level of professional autonomy among nurses working in Türkiye. However, the general view was that nurses’ level of professional autonomy was moderate.

One nurse said, *“I can evaluate the current situation as moderate. I cannot say that it is sufficient. Many factors are effective, as we have discussed before, but of course this does not change the result” (Nurse 8)*.

### Issues to be considered in the professional autonomy of nurses

Nurses stated that the issues to be considered when exhibiting professional autonomy were: the prioritization of career development; knowledge of professional rules, duties, and responsibilities; respect of the profession; provision of communication and cooperation; and the efficacy of professional organizations. Nurses emphasized that respect of and care for the profession are essential to professional development.

Regarding this subject, one nurse commented: *“First, you must care about your profession and try to work autonomously. You also need to conduct your professional duties with respect. This is particularly important in taking real responsibility for the work you do” (Nurse 4).*

### Individual professional autonomy

Nurses defined individual professional autonomy using two basic approaches: self-assessment of professional autonomy and the factors affecting the individual in exhibiting professional autonomy.

### Self-assessment of professional autonomy

Nurses gave different self-evaluations regarding professional autonomy, but the common view was that they found their level of professional autonomy to be moderate.

One nurse said that *“it [my professional autonomy] continues to improve, but I can say that it is at a moderate level right now” (Nurse 12).*

### Factors affecting professional autonomy

Nurses stated that their level of education and professional experience, prioritization of personal and professional development, awareness of professional autonomy, love of the profession, approach of management, presence of role models, quality of the team they work with, and motivation affected their professional autonomy, acknowledging its importance in their field.

One nurse commented: “I care about making my own decisions, being autonomous. I strive for this….Some of our colleagues don’t even think that the profession is independent. It’s a complete disaster” (Nurse 14).

### Autonomy regarding nursing roles

Nurses identified 13 different roles regarding their professional autonomy. These were: caregiving roles, training roles, research roles, administrative roles, decision-making roles, patient advocacy roles, communication and coordination roles, rehabilitative roles, comforter roles, therapeutic roles, career-enhancing roles, autonomy and responsibility roles, and consulting roles.

### The caregiver role

Nurses described autonomy in the caregiving role as the ability to independently perform nursing care practices, determine patients’ care needs, and provide patient-centered and holistic care. They emphasized the importance of independent decision-making in the nursing care process.

*“Performing the nursing process,”* said one nurse, *“that is, implementing nursing interventions, performing them independently…truly independently—that is autonomy in nursing care” (Nurse 1).*

### The trainer role

Nurses described autonomy related to the educational role as prioritizing professional education and development and being able to recognize and plan for patients’ educational needs.

One nurse commented: *“Many colleagues in the field do not provide adequate education to the patient. An autonomous nurse needs to be able to recognize the patient’s educational needs and plan for this” (Nurse 11).*

### The researcher role

Nurses described autonomy associated with the research role as identifying the research area, participating in multidisciplinary research, and following up on research results. They emphasized the importance of independently following the rapidly changing and developing literature.

On this topic, one nurse commented: *“When the issue is our role as researcher, nurses need to adapt and be able to catch up on the rapidly changing literature. Again, we need to follow the legal compliance of our practice” (Nurse 12).*

### The administrator role

Nurses described the autonomy associated with the manager role as managing patient processes, making effective decisions, advocating and guiding employees, and making decisions in accordance with legal regulations. They emphasized the role that nurse managers play in this respect, especially in terms of advocating for their colleagues and team members.

One nurse stated that *“a nurse manager must stand behind their team and have the capacity to act autonomously” (Nurse 14).*

### The decision-maker role

Nurses described their autonomy related to the decision-making role as the ability to design a work plan and ensure continuity, think multidimensionally, and make the right decisions.

*“Making decisions,”* said one nurse, *“especially in an important situation concerning the patient, is a reflection of autonomy in my opinion” (Nurse 1).*

### The patient advocate role

Nurses described their autonomy related to the patient advocate role as the ability to ensure that the patient is informed, prevent harm to the patient, protect patient rights, and ensure patient safety. They particularly highlighted the importance of preventing harm to vulnerable groups.

Regarding this, one nurse commented: *“If there are situations that we think will harm the patient in the practices we or others perform, we should bring this up and, if necessary, report it to the relevant authorities” (Nurse 17).*

### The communication and coordination role

Regarding autonomy related to nurses’ communication and coordination role, participants highlighted the importance of clear and effective communication with patients and the healthcare team.

One nurse commented: *“Of course, every nurse can make their own decisions, but we are a multidisciplinary professional group, so autonomy in our communication role means establishing good team communication” (Nurse 2).*

#### The rehabilitative role

Nurses described autonomy related to the rehabilitative role as the ability to provide a peaceful end-of-life period for patients and identify rehabilitation needs

*Concerning the rehabilitation role,”* said one nurse, *“being able to direct the patient to free resources or support should be among the independent functions of the nurse” (Nurse 16).*

### The comforter role

Concerning this role, nurses stated that patients should feel comfortable during treatment and care processes, both for the sake of recovery and the patient’s well-being. They emphasized that autonomous nursing interventions are necessary for patients who are stressed or in critical condition.

On this subject, one nurse commented: *“There may be conditions where the patient can be more physiologically, psychologically, and socially comfortable, but we need to be able to provide the patient with such conditions” (Nurse?).*

### The therapeutic role

Nurses described autonomy related to the therapeutic role as the ability to plan therapeutic interventions, implement treatment, and monitor treatment results.

One nurse stated that *“monitoring the effects of treatment and knowing how to do this is when the nurse is autonomous” (Nurse 8).*

### The career-enhancing role

Nurses described autonomy related to career development as the ability to follow up-to-date information, participate in scientific activities, pursue career opportunities, and attach importance to specialization.

On this subject, one nurse gave the following comment: *“Prioritizing both individual and professional development is a must. Being able to create career opportunities when necessary is especially important” (Nurse 7).*

### The autonomy and responsibility role

Nurses expressed that this role required making independent decisions, ensuring continuity of such decisions, encouraging nursing care visibility, and taking responsibility for decisions.

*I think autonomy in this role means acting professionally and being able to implement decisions and ensure their continuation (Nurse?).*

### The consultant role

Nurses stated that autonomy related to the counselor role required the ability to act independently, provide accurate information, and offer counseling and guidance to their colleagues and students. They emphasized the importance of being able to guide individuals not only in cases of illness but also in developing and maintaining health.

One nurse commented: *“When I say counseling, being a guide comes to mind. This is a very comprehensive job. It’s important to know the job well and be able to offer guidance to the patient” (Nurse 4).*

## Discussion

This study explored how nurses perceive and experience professional autonomy within their distinct professional roles. Participants defined autonomy primarily as acting in line with professional principles, laws, and job descriptions, while also emphasizing the importance of education, professional development, and effective regulations. Autonomy was expressed across caregiving, education, research, management, advocacy, and career development, reflecting the multifaceted and expanding scope of nursing practice [9, 10, 12].

Hall (2009) first described autonomy as independent professional decision-making, while more recent research has emphasized its connection with values, legal frameworks, and responsibilities [1, 7]. Consistent with Müller and Niessen [23], participants in this study highlighted autonomy as an indispensable aspect of professionalism. Autonomy was seen to increase with postgraduate education and career development, in line with findings by Erikmen and Vatan (2019) [24] and Dikmen et al. (2016) [25]. Similarly, Yıldırım and Alparslan [15] noted that autonomy enhances professional self-esteem, supporting our participants’ views on the link between professional growth and autonomy. These studies support our findings that professional autonomy is closely associated with professionalism, education, and regulatory frameworks.

Direct patient care was identified as the most prominent domain of autonomy, especially regarding decision-making within the nursing process and holistic care. This corresponds with previous studies emphasizing care as the central area of autonomous practice [2, 4, 8]. Our participants also stressed autonomy in planning patient education, keeping up with evidence, and contributing to research—dimensions highlighted by Kajander-Unkuri et al. (2018) [26] and Potter and Perry (2015) [27]. They emphasized that autonomy plays a crucial role in planning patient education tailored to individual needs and ensuring that educational content directly serves patients’ specific conditions. In this context, autonomy also enables nurses to identify practice gaps, design relevant research, and access and apply evidence-based knowledge in clinical decision-making. They further noted that autonomy in therapeutic and rehabilitative roles involves monitoring treatment outcomes and collaborating across disciplines to meet patients’ needs, which is consistent with findings by Farčić et al. [28].

Managerial and organizational factors were also found to be crucial. Participants emphasized that professional organizations, such as the Turkish Nurses Society—the main national body representing nurses in Türkiye—and other associations including the Turkish Nurse Managers Association and the Turkish Surgical Nurses Association, as well as international organizations such as the International Council of Nurses (ICN) and the American Nurses Association (ANA), are not sufficiently followed or actively engaged with by nurses. They also noted that, particularly at the national level, nurse managers do not provide adequate support to strengthen nurses’ professional autonomy. These perspectives align with evidence that professional associations and managerial support enhance autonomy and improve job satisfaction [6, 14, 29]. Pesut et al. (2023) [17] and Zuzelo (2024) [13] further underline the system-wide effects of autonomy, showing its links to quality of care, organizational commitment, and health outcomes. The lack of strong organizational structures was perceived by our participants as one of the most significant barriers to autonomy, echoing global findings.

By examining autonomy through the lens of distinct professional roles, this study provides a more concrete reflection of clinical practice than general discussions of autonomy. For instance, autonomy was expressed through independent decision-making in patient care, planning and delivering education, following and generating research, defending colleagues in managerial positions, advocating for patients’ rights, and creating new career opportunities. This role-based perspective reveals specific dimensions of autonomy that are often overlooked in broader analyses, offering novel insights into how autonomy is enacted in daily practice [21, 25].

These findings carry important implications for nursing practice and health policy. Strengthening professional autonomy requires addressing regulatory uncertainties, fostering continuous professional education, and empowering professional organizations. Nurse managers and policymakers share responsibility for creating supportive environments where autonomy can flourish. At the same time, nurses themselves must take active responsibility by engaging in lifelong learning, participating in professional associations, and consciously exercising autonomy in their roles [3, 25]. Such combined efforts are essential not only for professional development and job satisfaction but also for improving patient outcomes and ensuring more effective, patient-centered health systems worldwide.

## Limitations

This study has several limitations. First, the sample size was relatively small and limited to nurses working in Istanbul, which restricts the generalizability of the findings. Although maximum variation sampling was used to increase the diversity of participants, further research with larger and more diverse samples is needed. Second, the absence of male participants—despite efforts to recruit them—means that gendered perspectives on autonomy could not be fully captured. Given the growing number of male nurses internationally, this represents an area for future exploration. Finally, the study addressed autonomy across all professional roles, which provided breadth but limited in-depth analysis of each role. Future research should therefore focus on examining autonomy within specific professional roles in greater depth, to capture the unique challenges and opportunities associated with caregiving, education, management, and other domains of nursing practice.

## Conclusion

The findings suggest that strengthening professional autonomy in nursing requires a comprehensive, multi-level approach. This includes clarifying and harmonizing legal regulations, sustaining investment in education and career development, safeguarding opportunities for independent clinical judgment, and reinforcing the capacity and visibility of professional organizations. Equally important is the creation of supportive working environments that allow nurses to exercise autonomy in daily practice and to contribute actively to decision-making at institutional and policy levels. Developing autonomy, however, depends not only on systemic reforms but also on the active engagement of nurses themselves. Health managers and policymakers must provide enabling structures, while nurses must take responsibility through lifelong learning, participation in professional associations, and self-awareness of their professional roles. Such combined efforts will not only advance autonomy and strengthen professional identity but also help address professional challenges and contribute to more resilient, patient-centered health systems and improved patient outcomes.

### Implication and recommendation

This study underscores the importance of viewing professional autonomy through the lens of distinct nursing roles such as caregiving, education, management, and advocacy, each of which carries unique opportunities and challenges. For practice, strengthening role-specific autonomy enables nurses to make independent, evidence-informed decisions that improve patient-centered care and collaboration. For management, unit leaders should create environments that recognize role-based expertise, support participation in interdisciplinary decision-making, and foster conditions that sustain professional satisfaction. For policy, regulations and organizational frameworks should explicitly safeguard and expand nurses’ autonomy across their roles, while stronger professional organizations are needed to ensure that nurses’ voices are represented in institutional and national policy processes. Nurses themselves also carry responsibility for cultivating autonomy by engaging in lifelong learning, joining professional associations, and critically reflecting on their responsibilities. Finally, future research should focus on autonomy within specific professional roles and include larger, more diverse samples, including male nurses, to provide deeper insights and inform strategies that advance nursing practice and health system outcomes.

## Data Availability

The data that support the findings of this study are available from the corresponding author on reasonable request.

## Abbreviations

COREQ: Consolidated Criteria for Reporting Qualitative research
